# Exploring Medication Errors with Antipsychotics in Saudi Arabia: Insights from a Nationwide Analysis

**DOI:** 10.3390/healthcare13212705

**Published:** 2025-10-27

**Authors:** Ghadah H. Alshehri, Leena I. Al Awn, Salma M. Aldossari, Wafa S. Alluwaymi, Rashed A. Alghanim, Afnan S. Almordi, Reham F. Hettah, Sahar F. Almushaikah, Asma M. AlShahrani, Nouf T. Alshammri, Otilia J. F. Banji, Lamaa S. AlAmri, Nada A. Alsaleh, Badr G. Alghamdi

**Affiliations:** 1 Department of Pharmacy Practice, College of Pharmacy, Princess Nourah bint Abdulrahman University, Riyadh 11671, Saudi Arabia; naaalsaleh@pnu.edu.sa; 2 Pharmaceutical Care Department, King Abdulaziz Medical City, Riyadh 11426, Saudi Arabia; 3 Therapeutic Affairs Deputyship, Ministry of Health, Riyadh 12382, Saudi Arabia; 4 Department of Clinical Practice, College of Pharmacy, Jazan University, Jazan 45142, Saudi Arabia; 5 Pharmacy Practice Research Unit, College of Pharmacy, Jazan University, Jazan 45142, Saudi Arabia; 6 Pharmaceutical Care Department, King Khalid Hospital, Ministry of Health, Alkharj 16271, Saudi Arabia

**Keywords:** medication errors, antipsychotic drugs, Ministry of Health, Saudi Arabia

## Abstract

**Background/Objectives:** The objective of this study is to investigate the patterns and characteristics of medication errors (MEs) associated with antipsychotic medication use in hospitals affiliated with the Ministry of Health (MOH) in Saudi Arabia and to identify areas for improvement. **Methods:** A retrospective descriptive analysis of MEs associated with antipsychotic use was conducted using data collected from MOH-affiliated hospitals between April 2020 and September 2022. The data were analyzed descriptively to identify the factors underpinning unsafe antipsychotic use. **Results:** The sample period produced 35,077 reported MEs. Reports from the Western region contributed the highest error percentage, and MEs were reported more frequently in male (76.1%, *n* = 26,705) and adult (97.7%, *n* = 34,275) patients. Pharmacists reported MEs more often than other healthcare professionals (66.5%, *n* = 23,312). Most MEs (89.9%, *n* = 31,524) originated in the prescribing stage, with missing prescription information being the most frequently reported ME type (40.5%, *n* = 14,206). Atypical antipsychotics accounted for the greatest proportion of reports (79.3%, *n* = 27,811) compared to typical antipsychotics (20.7%, *n* = 7262). Most ME outcomes fell into Category B: The error occurred but did not reach the patient (56.4%, *n* = 19,794). Factors related to staffing or workflow accounted for 21.3% (*n* = 7467) of the reported errors, followed by a lack of policies in relation to antipsychotics prescribing and monitoring (20.5%; *n* = 7195). **Conclusions:** MEs in hospitals in Saudi Arabia frequently involve antipsychotic medications. This study identified important targets that may help reduce such risks in the future.

## 1. Introduction

Medication errors (MEs) contribute significantly to preventable harm and impact 3.3% of the population, sometimes causing life-threatening complications [1,2]. The National Coordinating Council for Medication Error Reporting and Prevention (NCC MERP) defines a medication error as “any preventable event that may cause or lead to inappropriate medication use or patient harm while the medication is controlled by the healthcare professional, patient, or consumer. Such events may relate to professional practice, healthcare products, procedures, and systems, including prescribing, order communication, product labelling, packaging, nomenclature, compounding, dispensing, distribution, administration, education, monitoring, and use” [3].

The risk of MEs may be higher in psychiatric hospitals due to the complexities associated with patient care and factors such as disorganized cognitive function, emotion, and behavior [4,5]. Antipsychotic medications are one of three major classes that act on the central nervous system reported to be involved with MEs [6,7]. Antipsychotic medications are prescribed for schizophrenia, bipolar disorders, and schizoaffective disorders, but their use has expanded to include the treatment of depression, insomnia, autism spectrum disorders, and behavioral symptoms of dementia [8,9,10,11,12]. The wider application and off-label use of these medications have led to a significant increase in users, which raises concerns about the risk of MEs [13].

Several factors increase the likelihood of MEs associated with antipsychotic drugs, including prolonged action durations and extended treatment periods related to the chronicity of the health conditions they are treating [14]. Additionally, other factors, such as parenteral administration routes [15], polypharmacy [16,17,18], the use of high-risk medications (e.g., clozapine) [19,20], and high doses intended to tranquilize the patient rapidly, [21], also contribute to the risk of ME occurrence.

MEs in psychiatric hospitals are common and account for 5.7 to 88.8 MEs per 100 admissions [22] in which they are reported to be contributed by a variety of potential factors, including patient-, medication-, and hospital-related factors [23]. Historically, the ME reporting system has been the primary method of collecting MEs within healthcare organizations [24,25]. Many countries have established national reporting systems for this purpose, such as the National Reporting and Learning System (NRLS) in England and Wales [26], the British Columbia Patient Safety and Learning System in Canada [27], and the Ministry of Health (MOH)-ME reporting system in Saudi Arabia [28]. Such reporting systems are considered an important source of information that helps identify patterns and contributory factors of MEs, which are essential prerequisites for developing interventions that can be applied in practice [29]. In the United States, data from the National Poison Data System revealed a sharp rise in MEs between 2000 and 2012 due to the use of antipsychotics [30]. A study analyzing ME data from the NRLS used in England and Wales identified that a significant proportion of ME reports (10.4%) involved antipsychotic medications [31]. Similarly, in Thailand, a 10-year retrospective analysis of the national ME database found that 8.9% of all MEs involved antipsychotic medication [32]. In Saudi Arabia, an analysis of 23,355 ME reports retrieved from 21 psychiatric hospitals revealed antipsychotic medications to be involved in 7769 cases (59.4%), which indicates that antipsychotics are the drug class most frequently implicated in this setting [7]. In general hospitals, the factors related to patient harm from MEs are complex, including those related to healthcare providers [33,34], the healthcare system [35,36], and patient characteristics [16,23,37,38]. Several studies have highlighted that the risk of harm arising from MEs is common in psychiatric settings [6,22,31]. These findings reveal an urgent need to prioritize and detect MEs in these settings [23].

In Saudi Arabia, the MOH is the primary provider of mental health services, which it delivers through the regionally structured General Administration for Mental Health and Social Services [39,40]. Each region has hospitals that provide inpatient, outpatient, and emergency services alongside specialized facilities for children and adolescents [41] and additional support from private providers, community clinics, and primary healthcare centers. In the interest of improving medication safety in psychiatric treatment, the MOH has launched several initiatives to ensure equal, accessible, and integrated healthcare services for patients with psychiatric disorders [42]. More specifically, the MOH’s General Department of Pharmaceutical Care has supported efforts to detect MEs using ME reporting system to better understand the nature of MEs reported in MOH-affiliated hospitals, identify learning opportunities, and generate recommendations to improve medication safety [28]. This goal aligns with the World Health Organization’s third global initiative—Medication Without Harm [43]—as well as Saudi Arabia’s Vision 2030, which lists ‘enhancing the quality and safety of the healthcare system’ among the initiative’s priorities [44].

The reported prevalence of schizophrenia, schizotypal, and delusional disorders treated in MOH mental health facilities was 8624 inpatients and 141,775 outpatients [45]. Schizophrenia is also recognized as the third leading global cause of disability and reduced life expectancy [46,47], which points to its significance as a major public concern. Accordingly, it is necessary to conduct research across multiple hospitals in Saudi Arabia to assess the pattern and contributory factors of MEs associated with antipsychotic use. Therefore, this study aims to identify the ME patterns associated with antipsychotic medication use and to determine factors that might support the development of strategies to mitigate risk and improve patient outcomes in psychiatric settings.

## 2. Methods

### 2.1. Study Design

The study adopted a cross-sectional design to assess MEs associated with the use of antipsychotic medications in MOH-affiliated hospitals in Saudi Arabia from April 2020 to September 2022. Reports prior to April 2020 were excluded due to system updates that created inconsistencies with subsequent data, thereby undermining the reliability of the data coding and analysis. Data from private and non-MOH hospitals were not included because these institutions use separate internal reporting systems that do not communicate with the MOH-ME reporting system. The study was conducted and reported in accordance with ‘The REporting of studies Conducted using Observational Routinely collected health Data Statement’ and the completed RECORD checklist is included as Supplementary Material, Table S1 [48].

### 2.2. Data Source

Data were extracted from the National ME Reporting System, a standardized platform developed by the MOH’s General Administration of Pharmaceutical Care. The system was established to detect reports of MEs from MOH-affiliated hospitals across Saudi Arabia and is the largest national database of its kind. Reporting MEs is mandatory for all MOH hospitals and follows a structured format to ensure the capture of all information related to MEs. Over the years, the system has been continuously refined to improve data quality and standardization, with reporting arrangements coordinated and overseen by the MOH [28]. Healthcare providers in MOH hospitals submit ME reports through their local reporting systems for investigation and analysis. These reports are then transmitted to the MOH’s General Administration of Pharmaceutical Care for central review. Each ME report includes patient demographics (i.e., age and gender), the region in which the MEs occurred in Saudi Arabia (i.e., Central, Western, Eastern, Northern, and Southern Regions), reporter profession, error type (e.g., wrong drug, wrong dose), error stage (i.e., prescribing, transcribing, dispensing, administration, or monitoring), factors identified by the reporter to increase the likelihood of ME occurrence, and ME outcome according to NCC MERP ME index (see Table 1) [3]. Although several frameworks have been developed for ME classification [49,50], the MOH uses the NCC MERP framework, which was adopted for use in this study as it provides a standardized and validated internationally recognized method for classifying MEs [3].

### 2.3. Data Screening and Extraction

ME reports that listed “antipsychotic medications” under the ME reporting system’s “medication name/category” field were retrieved and included in this study. Three of the study’s authors independently classified antipsychotic medications as “typical” or “atypical” in accordance with the MOH’s Drug Formulary categorizations, ensuring full adherence to the official MOH classification system to maintain consistency with the national reporting framework. They also independently coded existing MOH variables, such as patient demographics, ME type and stage, reporter profession, contributory factor, and ME outcome. Supplementary Table S2 presents the list of antipsychotic drugs included in the analysis.

Any missing values in the database variables were retained and categorized as “Not reported.” The action plan reported for each ME was not included in the analysis, as these are beyond the scope of this study. All ME reports included in this study were fully anonymized to maintain patient and reporter confidentiality.

### 2.4. Data Analysis

Descriptive analyses were conducted to summarize the main characteristics of antipsychotic-related MEs, including the frequency of MEs over time, patient age, patient gender, reporter profession, ME type, ME stage, ME outcome, and contributing factors, all of which were presented as numbers and percentages. Cross-tabulations were used to explore the association between these variables, including ME stages, error types, drug class (typical or atypical), and their associated outcomes. This association was tested using the chi-square test, with statistical significance set at *p* < 0.05. The analyses were conducted using SPSS Version 30 (2024).

## 3. Results

**Figure 1:**
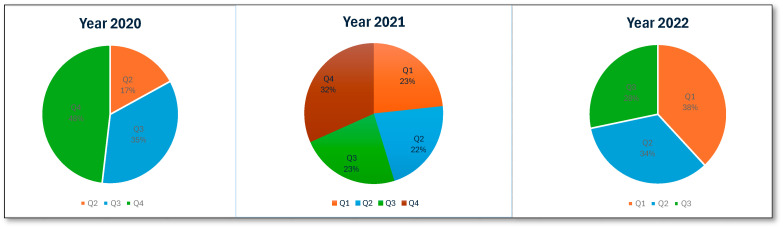
Number of antipsychotic-related MEs per quarter.

An analysis of ME reports related to patients receiving antipsychotic medications in Saudi Arabia between 2020 and 2022 identified 35,077 errors. The total number of MEs revealed a higher number in 2021 (45.1%, *n* = 15,819), while a significantly lower number was observed in 2020 (22%, *n* = 7707). Figure 1 presents the number of MEs per quarter. MEs were reported more frequently in male patients (76.1%, 26,705) than in female patients. The highest proportion of MEs was reported in adult patients (97.7%, *n* = 34,275). An examination of the reporting pattern among healthcare professionals showed that pharmacists and pharmacy assistants were most frequently involved in ME reporting: 66.5% (*n* = 23,312) and 21.8% (*n* = 7651), respectively. Physicians and patients less frequently report MEs, accounting for 3.6% (*n* = 1234) of the total reports, while nurses accounted for 5.2% (*n* = 1807) of the reported errors. An examination of the number of MEs reported in the five regions of Saudi Arabia revealed that the Western region accounted for the highest percentage of reports (32.8%, *n* = 11,496). The proportion of reports from other regions was much lower, with the Central region accounting for 18.5% (*n* = 6477) and the Northern, Southern, and Eastern regions reporting figures similar to or lower than the Central region. Regarding the stages at which MEs occurred, the prescribing stage accounted for the highest percentage (89.9%, *n* = 31,524), the dispensing stage accounted for 5.8% (*n* = 2047), and the transcribing and monitoring stages contributed minimally. The most negligible errors were reported at the administration stage (0.8%, *n* = 285). Table 2 presents the summary statistics for antipsychotic-related MEs.

**Figure 2:**
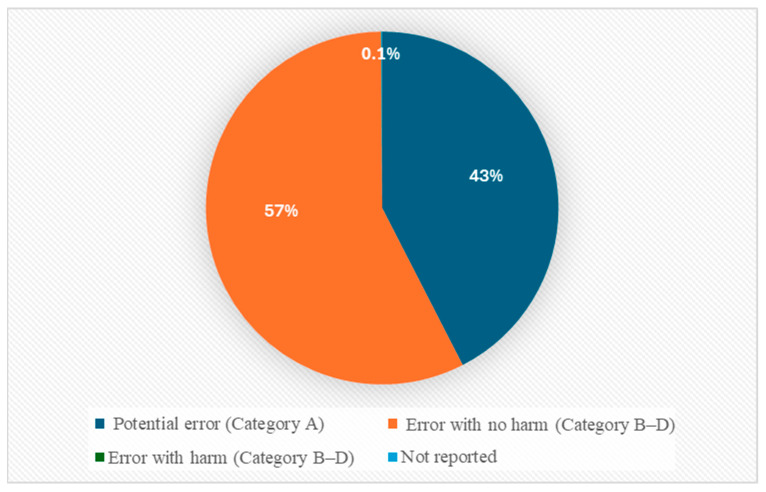
ME categorization based on NCC MERP index.

Most of the reported antipsychotic-related MEs were recorded in Categories A and B, representing potential error and MEs that did not reach patients (42.5%, *n* = 14,891 and 56.4%, *n* = 19,794, respectively). A smaller number were reported in Category C (MEs reaching the patient without harm: 0.9%, *n* = 326) and Category D (MEs requiring monitoring: 0.1%, *n* = 26). Few MEs were reported as involving actual harm (Categories E–I), which accounted for 0.04% (*n* = 13) of the reports. Overall, 98.9% of all reports represented potential harm (Categories A–D), while just 0.04% (*n* = 13) were linked to actual harm (Categories E and F). The remaining reports (0.1%, *n* = 27) did not provide any data on ME-related outcomes. No ME reports were classified as Category G or H (see Figure 2).

A cross-tabulation analysis between ME stage and outcome was also conducted. Most antipsychotic-related MEs across all stages were reported to be associated with nonharmful outcomes. Prescribing MEs accounted for 31,487 reports (99.9%) that did not result in patient harm, with 12 MEs resulting in harmful outcomes. MEs in the dispensing (*n* = 2046; 100%), transcribing (*n* = 839; 100%), administration (*n* = 285; 100%), and monitoring stages (*n* = 354; 99.7%) were reported as having nonharmful outcomes. No significant association between ME stage and outcome was found using the chi-square test (χ^2^ = 5.236, *p* = 0.875) (see Table 3).

The number and percentage of MEs by type are summarized in Table 4. Most MEs were reported to be related to a lack of vital details, such as the patient’s age, allergies, and diagnostic information, collectively referred to as prescription information. Oversights in this category contributed to 40.5% (*n* = 14,206) of the identified MEs. Other MEs were reported as being related to the absence or incorrect documentation of dosage information (14.3%, *n* = 5033), with the remaining ME types contributing to 13.9% (*n* = 4874). A little over 15% (*n* = 5550) of the MEs were reported to be linked to incorrect dosing and frequency of administration or incorrect/unclear/missing medication names.

Of the total number of antipsychotic medications involved with MEs, around 80% (*n* = 27,811) of MEs were atypical. Olanzapine (33.1%; *n* = 11,607) and quetiapine (19.4%; 6804) were the most frequently reported drugs. Of the 20.7% (*n* = 7262) of reported MEs involving typical antipsychotics, the most frequently reported drugs were haloperidol (14.5%; 5098) and chlorpromazine (2.9%; 1025). The remining report was reported to involve both classes (*n* = 4). The top five reported typical and atypical antipsychotic drugs and their associated outcomes are presented in Table 5. No significant difference was observed between the antipsychotic drug type and ME outcome (χ^2^ = 16.241, *p* = 0.981).

Table 6 presents the factors that contributed to reported MEs. The three leading factors were staffing or workflow-related (21.3%, *n* = 7467), followed by the absence of policies related to prescribing and monitoring (20.5%, *n* = 7195) and environmental factors (16.7%, *n* = 5875). Poor handwriting (11%, *n* = 3876) and staff inexperience (9.2%, *n* = 3217) were also significant contributors. Issues with electronic systems, incorrect labeling, look-alike medications, and prohibited abbreviations contributed to a lesser extent. The other minor contributing factors were patient-related, such as those involving sound-alike medications and storage arrangements.

## 4. Discussion

This retrospective study assessed the pattern of MEs related to antipsychotic use in MOH-affiliated hospitals in Saudi Arabia. The total number of MEs related to antipsychotic use varied across the year; however, the higher number of ME reports from 2021 (*n* = 15,819) and 2022 (*n* = 11,551) compared to 2020 (*n* = 7707) likely reflects the inclusion of data over a full year (12 months) compared to the nine months (April to December) captured for 2020. Thus, the variation might be due to the timeframes included rather than a real temporal trend. Additionally, the COVID-19 pandemic might have influenced reporting rates [51,52,53]. While published studies showed inconsistent effects on reporting, a study based on Saudi Arabia specifically reported an increase from 1.5 to 19 MEs per 100 medication orders during the COVID-19 pandemic [54].

The data from this study revealed that MEs occurred most frequently in relation to male and adult patients. Antipsychotic medication use has been reported to increase with age [40], which may account, in part, for the higher rates of MEs observed in the adult population. The findings of our study are consistent with previous research reporting a higher percentage of MEs in males treated with clozapine [20]. Moreover, the fact that males are at a higher risk of developing schizophrenia than females [55] suggests that the former may be more susceptible to MEs due to the increased likelihood of being treated with medication. On the other hand, the higher proportion of adult males reported to be involved in MEs likely reflects the types of hospitals contributing to the MOH’s ME reporting system, as women and children may not be fully represented in this reporting system. This pattern may also be influenced by reporting bias related to errors involving these groups of patients [56]. A considerable regional disparity was also found in the reporting of antipsychotic-related MEs. Approximately one-third of the reported MEs originated in the Western region, while the remaining two-thirds were distributed across the other four. Future research could explore the potential factors for this regional disparity. Our findings suggest that pharmacists are the most likely to report MEs, while physicians and patients make far fewer reports. The fact that pharmacists are the most frequently involved in ME reporting has been substantiated in studies conducted in Saudi Arabia and other parts of the world [4,28,57].

Our study highlights the extent to which atypical antipsychotics are involved in MEs, specifically that they are reported more frequently than MEs associated with typical antipsychotics. These results are unsurprising, since atypical antipsychotic drugs are used more frequently than typical antipsychotics due to their efficacy in treating a wide range of negative schizophrenia symptoms and because they carry a lower risk of motor side effects, such as extrapyramidal symptoms and tardive dyskinesia, which negatively impact patients’ quality of life and may subsequently reduce their long-term treatment adherence [58,59,60]. Moreover, atypical antipsychotics, such as clozapine and risperidone, have been associated with potentially hazardous prescribing while treating psychiatric disorders [61,62]. Therefore, atypical antipsychotics should be prioritized to reduce the risk of MEs associated with their use.

The results of our study suggest that most MEs related to antipsychotic medication use throughout Saudi Arabia are caused by prescription errors. Approximately two-fifths of the prescriptions lacked complete details, which reveals a gap in the documentation process. Around 15% of the MEs were attributed to incomplete or missing dosage details, and a little over 20% omitted crucial medication information such as the name, frequency, and treatment duration. Other studies have shown that MEs are frequently reported in mental health settings and during prescription ordering or transcribing [6,63,64,65]. For instance, a 2007 study by Rothschild et al. reported an error rate of 68% related to prescription orders [66], and a study conducted in a French psychiatric hospital found that PEs accounted for 55.3% of the total number of MEs committed [4]. Further evidence from a UK that used Clinical Practice Research Datalink (CPRD) data has revealed a notable link between mental health prescribing patterns and patient safety outcomes, which supports our findings that antipsychotics, particularly in the prescribing stage, should take priority when implementing interventions to ensure safe medication use [67]. Other published studies conducted in Saudi Arabia showed that MEs are common even in nonpsychiatric settings, such as in primary care settings, which account for one-fifth of all reported MEs [68]. A systematic review examining MEs in the Middle East identified PEs as the most common ME type [5,55]. Another study conducted in secondary healthcare centers in Kuwait found that 62% of MEs are related to PEs [69]. However, it is important to note that while PEs represent a higher percentage of the reported errors, administration (0.8%) and monitoring (1.0%) accounted for only small proportions. This pattern should be interpreted cautiously, as this is likely to reflect the reporting pattern rather than genuinely low risk. Inconsistent capture of these errors across sites may introduce reporting bias and should be considered when interpreting these results [56].

Our findings demonstrate that the vast majority of antipsychotic-related MEs were classified as near misses or errors without patient impact (Categories A–D) and that only a very small proportion were associated with actual harm (Categories E–I). This distribution is consistent with the nature of reporting systems, which tend to capture near-miss and no-harm events rather than harmful outcomes. The predominance of near-miss reporting highlights opportunities for early intervention to prevent future patient harm [70].

Our study identified three primary factors that contribute to MEs, including staffing, workflow, and the absence of suitable policies related to antipsychotic medication use, a finding that aligns with the results of other studies conducted in psychiatric settings [7,16,20,31]. Increased patient turnover increases the workload, which may not be matched by the existing workforce and may, in turn, enhance the likelihood of errors. This particular issue has been identified in studies whose authors found that overburdened staff cannot work diligently, which compromises the quality of care [71]. Moreover, environmental factors, such as overly crowded workspaces, distracting noises, and inadequate illumination, also contributed to MEs. These findings are consistent with those reported by Keers et al., who highlighted the increased chance of slip-ups or lapses in workplaces where distractions are present [16,38].

### Implications for Practice and Future Research

Several interventions have been recommended to reduce the risk of MEs in hospitals, such as staff education, the integration of technology in the medication use process (e.g., Pharmacist-Led Information Technology Intervention for Medication Errors [PINCER]) [72], and the involvement of ward-based clinical pharmacists [73,74,75]. A targeted implementation study could evaluate both the effectiveness and feasibility in preventing antipsychotic-related MEs. The predominance of MEs in the prescribing stage, as well as the high percentage of reported near-miss and no-harm MEs, suggest an opportunity to focus on strengthening early safeguards within the medication use process, specifically by implementing structured identification and monitoring of unsafe prescribing practices. The use of prescribing safety indicators (PISs) has been found to be effective in psychiatric settings and offers an evidence-based approach for enhancing antipsychotic prescribing in psychiatric hospitals in Saudi Arabia [61].

At the system level, this study highlights the importance of maintaining the MOH-ME reporting system to support ongoing improvements in the safe use of antipsychotic medications. Future developments might focus on expanding reporting system capacities to allow the documentation of multiple ME types and contributory factors in individual ME reports to better reflect the complexity of the reported MEs. Additionally, refining category definitions—for example, differentiating between “incorrect/unclear/missing dose” and “dose omission”—might facilitate the targeted prevention of specific ME types. In particular, the “Other” option, which comprised 13.9% of reports and represented MEs that could not be classified using the predefined MOH-ME type classifications, presents a valuable opportunity for future system improvement. Future research is warranted to investigate the root cause of antipsychotic-related MEs using qualitative approaches, such as Reason’s Model of Accident Causation [16,76,77], that might shed light on the contextual factors involved in MEs. Additionally, interventional studies are warranted such as pharmacist-led review, electronic prescribing and medicine administration, or educational initiatives aimed at reducing antipsychotic-related MEs [78,79].

This study has several strengths. First, it presents a large-scale investigation of antipsychotic-related MEs in the Middle East and provides novel insights into medication safety in this regional context. Second, a dataset of 35,077 reports was sufficient to explore subgroup patterns across demographics, regions, and antipsychotic drug classes. Finally, applying the NCC MERP classification framework facilitated comprehensive ME outcome categorization that facilitated meaningful comparisons with international studies. Nevertheless, this study has several limitations. First, due to the inherent limitations of all voluntary ME reporting systems, MEs are likely to be underreported, and the data reflects reporting patterns (i.e., differences in infrastructure, staffing, or reporting culture) rather than the actual occurrence of MEs. Second, the lack of denominator data, such as total prescriptions and number of patient-days, limited the ability to calculate ME rates. Future studies are encouraged to collect denominator data to support more meaningful comparisons with national and international studies. Third, the ME outcome assessment was classified using the NCC MERP framework, which reflects the reporter’s perception of harm rather than actual clinical consequences. Additionally, the study period coincided with the COVID-19 pandemic, which may have influenced ME reporting pattern due to changes in healthcare delivery processes, staffing, and workload during that period. Moreover, our study employed the MOH classification system, with no reclassification conducted by the research team, which makes interrater reliability checks unnecessary; however, while this approach supports consistency, the potential for misclassification cannot be ruled out. Finally, the database does not capture information related to patients’ underlying conditions and comorbidities, which limit interpretations based on the clinical context. Future system enhancements would allow reporters to capture the complexity of antipsychotic-related MEs and better support the development of targeted future safety interventions.

## 5. Conclusions

This is the first national study of patterns and contributory factors of MEs related to antipsychotic use in MOH-affiliated hospitals in Saudi Arabia. The study found that most MEs originated in the prescribing and dispensing stages, with MEs related to drug doses and prescription information being the most frequently observed. Regarding the potential contributory factors associated with antipsychotic use, we identified staff shortages and lack of policies among the most frequently reported. To improve safety, targeted training for high-risk antipsychotics, pharmacist-led intervention and computerized prescribing with electronic medication administration could be considered to ensure safe antipsychotic medication use.

## Figures and Tables

**Table 1:** The NCC MERP Index for Categorizing ME Outcomes.

Outcome Category	Definition
Category A	Circumstances or events that have the capacity to cause error
Category B	An error occurred but the error did not reach the patient (an “error of omission” does reach the patient)
Category C	An error occurred that reached the patient, but did not cause patient harm
Category D	An error occurred that reached the patient and required monitoring to confirm that it resulted in no harm to the patient and/or required intervention to preclude harm
Category E	An error occurred that may have contributed to or resulted in temporary harm to the patient and required intervention
Category F	An error occurred that may have contributed to or resulted in temporary harm to the patient and required initial or prolonged hospitalization
Category G	An error occurred that resulted in permanent patient harm
Category H	An error occurred that resulted in a near-death event
Category I	An error occurred that resulted in patient death

**Table 2:** ME Dataset Descriptive Statistics.

ME Characteristics	Frequency (*n*)	Percentage (%)
Reporting Year		
2020	7707	22.0
2021	15,819	45.1
2022	11,551	32.9
Total	35,077	100.0
Gender	Frequency (*n*)	Percentage (%)
Male	26,705	76.1
Female	8368	23.9
Not reported	4	0.0
Total	35,077	100.0
Age	Frequency (*n*)	Percentage (%)
Child (1 day–13 years old)	483	1.4
Adolescent (13–18 years old)	313	0.9
Adult (>18 years)	34,275	97.7
Not reported	6	0.0
Total	35,077	100.0
Region	Frequency (*n*)	Percentage (%)
Western	11,496	32.8
Central	6477	18.5
Northern	5817	16.6
Southern	5749	16.4
Eastern	5522	15.7
Not reported	16	0.0
Total	35,077	100.0
Reporter Profession	Frequency (*n*)	Percentage (%)
Pharmacist	23,312	66.5
Pharmacist assistant	7651	21.8
Nurse	1807	5.2
Physician	1040	3.0
Patient/caregiver	194	0.6
Not reported	1067	3.0
Other	6	0.0
Total	35,077	100.0
Stage of error	Frequency (*n*)	Percentage (%)
Prescribing	31,524	89.9
Dispensing	2047	5.8
Transcribing	839	2.4
Monitoring	355	1.0
Administration	285	0.8
Not reported	27	0.1
Total	35,077	100.0

**Table 3:** Antipsychotic-Related MEs by Stage and Outcome.

Stage of Error	Nonharmful Outcome (*n*, %)	Harmful Outcome (*n*, %)	Not Reported (*n*, %)	Total (*n*)
Prescribing	31,487 (99.9%)	12 (0.0%)	25 (0.1%)	31,524
Dispensing	2046 (100%)	1 (0.0%)	0 (0.0%)	2047
Transcribing	839 (100%)	0 (0.0%)	0 (0.0%)	839
Monitoring	354 (99.7%)	0 (0.0%)	1 (0.3%)	355
Administration	285 (100%)	0 (0.0%)	0 (0.0%)	285
Not reported	27 (100%)	0 (0.0%)	0 (0.0%)	27
Total	35,038 (99.9%)	13 (0.0%)	25 (0.1%)	35,077

Nonharmful outcome (Categories A–D), Harmful outcome (Categories E–I), *p*-values derived from Pearson chi-square test (*p* = 0.875).

**Table 4:** Descriptive analysis of ME dataset by ME type.

Error Types	Frequency (*n*)	Percent (%)
Missing prescription information ^a^	14,206	40.5
Incorrect/unclear/missing dose	5033	14.3
Incorrect/unclear/missing frequency	3007	8.6
Incorrect/unclear/missing medication name	2543	7.2
Incorrect/unclear/missing duration	1968	5.6
Duplicate therapy	889	2.5
Drug dose omission	553	1.6
Incorrect/unclear/missing route of administration	510	1.5
Error related to dosage form	496	1.4
Incorrect/unclear/missing indication	298	0.8
Out of privilege ^b^	173	0.5
Missing medication history/labs	170	0.5
Drug–drug interaction	165	0.5
Hazardous situation	133	0.4
Error related to medication storage	39	0.1
Drug contraindications	10	0.0
Drug disease interaction	8	0.0
Other	4874	13.9
Not reported	2	0.0
Total	35,077	100.0

^a^ (e.g., patient name, medical record number, age, weight, allergy, diagnosis); ^b^ prescribing drug outside the provider’s authorized scope.

**Table 5:** Top Five Typical and Atypical Antipsychotics and Their Associated Outcomes.

Type	Medication	Nonharmful Outcome *n* (%)	Harmful Outcome *n* (%)	Not Reported *n* (%)	Total (*n*)
Atypical antipsychotics	Olanzapine	11,595 (99.9%)	3 (0.0%)	9 (0.1%)	11,607
	Quetiapine	6794 (99.9%)	4 (0.1%)	6 (0.1%)	6804
	Risperidone	4670 (99.9%)	2 (0.0%)	3 (0.1%)	4675
	Aripiprazole	1830 (99.8%)	3 (0.0%)	0 (0.0%)	1833
	Amisulpride	1336 (99.9%)	0 (0.0%)	1 (0.1%)	1337
Subtotal		26,225	12	19	26,256
Typical antipsychotics	Haloperidol	5092 (99.9%)	1 (0.0%)	5 (0.1%)	5098
	Chlorpromazine	1024 (99.9%)	0 (0.0%)	1 (0.1%)	1025
	Flupentixol	421 (100%)	0 (0.0%)	0 (0.0%)	421
	Trifluoperazine	368 (99.7%)	0 (0.0%)	1 (0.3%)	369
	Zuclopenthixol	320 (100%)	0 (0.0%)	0 (0.0%)	320
Subtotal		7225	1	7	7233
Total (All Drugs)		33,450	13	26	33,489

Nonharmful outcome (Categories A–D), Harmful outcome (Categories E–I).

**Table 6:** Frequency of potential contributory factors in reported MEs.

Potential Contributory Factors	Frequency (*n*)	Percentage (%)
Staffing or workflow-related factors ^a^	7467	21.3
Lack of policy	7195	20.5
Environmental factors ^b^	5875	16.7
Poor handwriting	3876	11
Lack of staff experience	3217	9.2
Problem related to electronic system	1940	5.5
Incorrect labeling	644	1.8
Attitude-related factors ^c^	360	1
Look-alike medications	350	1
Prohibited abbreviation ^d^	303	0.9
Patient-related factors ^e^	236	0.7
Sound-alike medications	177	0.5
Stock arrangement/storage problem	98	0.3
Other	3315	9.5
Not reported	24	0.1
Total	35,077	100

^a^ Staff shortage; ^b^ lighting, noise, interruption, and small or crowded working area; ^c^ errors linked to human behavior involving staff, patients, and caregivers; ^d^ use of nonstandard abbreviation; ^e^ noncompliance.

## Data Availability

The data that support the findings of this study are available from the Ministry of Health. Restrictions apply to the availability of these data, which were used under the license for this study. Data are available from the author(s) with the permission of the Ministry of Health.

## Supplementary Materials

The following supporting information can be downloaded at: https://www.mdpi.com/article/10.3390/healthcare13212705/s1, Table S1: RECORD Checklist; Table S2: List of antipsychotic drugs included in the analysis. Reference [80] is cited in the supplementary materials.

## References

[1] World Health Organization (2013). Mental Health Action Plan 2013–2020.

[2] Hodkinson A., Tyler N., Ashcroft D.M., Keers R.N., Khan K., Phipps D., Abuzour A., Bower P., Avery A., Campbell S. (2020). Preventable medication harm across health care settings: A systematic review and meta-analysis. BMC Med..

[3] National Coordinating Council for Medication Error Reporting and Prevention (NCCMERP) Medication Error Index. https://www.nccmerp.org/sites/default/files/index-color-2021-draft-change-10-2022.pdf.

[4] Lebas R., Calvet B., Schadler L., Preux P.-M., Laroche M.-L. (2024). Relationships between medications used in a mental health hospital and types of medication errors: A cross-sectional study over an 8-year period. Res. Soc. Adm. Pharm..

[5] Shehata Z.H.A., Sabri N.A., Elmelegy A.A. (2016). Descriptive analysis of medication errors reported to the Egyptian national online reporting system during six months. J. Am. Med. Inform. Assoc..

[6] Alshehri G.H., Ashcroft D.M., Nguyen J., Hann M., Jones R., Seaton K., Newton G., Keers R.N. (2021). Prevalence, nature, severity and preventability of adverse drug events in mental health settings: Findings from the MedicAtion relateD harm in mEntal health hospitals (MADE) Study. Drug Saf..

[7] Alenzi K.A., Alsheikh M.Y., Alsuhaibani D.S., Alatawi Y., Alshammari T.M. (2024). Medication Errors in Psychiatric Hospitals: A Nationwide Real-World Evidence Study in Saudi Arabia. Pharmaceuticals.

[8] Højlund M., Pottegård A., Johnsen E., Kroken R.A., Reutfors J., Munk-Jørgensen P., Correll C.U. (2019). Trends in utilization and dosing of antipsychotic drugs in Scandinavia: Comparison of 2006 and 2016. Br. J. Clin. Pharmacol..

[9] Marston L., Nazareth I., Petersen I., Walters K., Osborn D.P. (2014). Prescribing of antipsychotics in UK primary care: A cohort study. BMJ Open.

[10] Carton L., Cottencin O., Lapeyre-Mestre M., A Geoffroy P., Favre J., Simon N., Bordet R., Rolland B. (2015). Off-label prescribing of antipsychotics in adults, children and elderly individuals: A systematic review of recent prescription trends. Curr. Pharm. Des..

[11] Jobski K., Höfer J., Hoffmann F., Bachmann C. (2017). Use of psychotropic drugs in patients with autism spectrum disorders: A systematic review. Acta Psychiatr. Scand..

[12] Mok P.L., Carr M.J., Guthrie B., Morales D.R., Sheikh A., Elliott R.A., Camacho E.M., Van Staa T., Avery A.J., Ashcroft D.M. (2024). Multiple adverse outcomes associated with antipsychotic use in people with dementia: Population based matched cohort study. BMJ.

[13] Pirhonen E., Haapea M., Rautio N., Nordström T., Turpeinen M., Laatikainen O., Koponen H., Silvan J., Miettunen J., Jääskeläinen E. (2022). Characteristics and predictors of off-label use of antipsychotics in general population sample. Acta Psychiatr. Scand..

[14] Correll C.U., Rubio J.M., Kane J.M. (2018). What is the risk-benefit ratio of long-term antipsychotic treatment in people with schizophrenia?. World Psychiatry.

[15] Wang D., Schneider-Thoma J., Siafis S., Qin M., Wu H., Zhu Y., Davis J.M., Priller J., Leucht S. (2024). Efficacy, acceptability and side-effects of oral versus long-acting-injectables antipsychotics: Systematic review and network meta-analysis. Eur. Neuropsychopharmacol..

[16] Keers R.N., Plácido M., Bennett K., Clayton K., Brown P., Ashcroft D.M. (2018). What causes medication administration errors in a mental health hospital? A qualitative study with nursing staff. PLoS ONE.

[17] Stassen H., Bachmann S., Bridler R., Cattapan K., Herzig D., Schneeberger A., Seifritz E. (2022). Detailing the effects of polypharmacy in psychiatry: Longitudinal study of 320 patients hospitalized for depression or schizophrenia. Eur. Arch. Psychiatry Clin. Neurosci..

[18] Lelliott P., Paton C., Harrington M., Konsolaki M., Sensky T., Okocha C. (2002). The influence of patient variables on polypharmacy and combined high dose of antipsychotic drugs prescribed for in-patients. Psychiatr. Bull..

[19] Dabba K., Elswood M., Ameer A., Gerrett D., Maidment I. (2019). A mixed methods analysis of clozapine errors reported to the National reporting and learning system. Pharmacoepidemiol. Drug Saf..

[20] AlAmri L.S., Alluwaymi W.S., Alghamdi B.G., Alghanim R.A., Almordi A.S., Hettah R.F., Almushaikah S.F., AlShahrani A.M., Alshammri N.T., Aldossari S.M. (2024). Characteristics and causes of reported clozapine-related medication errors: Analysis of the Ministry of Health database in Saudi Arabia. Int. J. Clin. Pharm..

[21] Shahpesandy H., Tye N., Hegarty A., Czechovska J., Kwentoh M.L., Wood A. (2015). Rapid tranquillisation of acutely disturbed and violent patients: A retrospective cohort examination of 24 patients on a psychiatric intensive care unit. J. Psychiatr. Intensive Care.

[22] Alshehri G.H., Keers R.N., Ashcroft D.M. (2017). Frequency and nature of medication errors and adverse drug events in mental health hospitals: A systematic review. Drug Saf..

[23] Alshaikhmubarak F.Q., Keers R.N., Lewis P.J. (2023). Potential risk factors of drug-related problems in hospital-based mental health units: A systematic review. Drug Saf..

[24] Mann K., Rothschild J.M., Keohane C.A., Chu J.A., Bates D.W. (2008). Adverse drug events and medication errors in psychiatry: Methodological issues regarding identification and classification. World J. Biol. Psychiatry.

[25] Gandhi T.K., Seger D.L., Bates D.W. (2000). Identifying drug safety issues: From research to practice. Int. J. Qual. Health Care.

[26] Cousins D.H., Gerrett D., Warner B. (2012). A review of medication incidents reported to the National Reporting and Learning System in England and Wales over 6 years (2005–2010). Br. J. Clin. Pharmacol..

[27] British Columbia Patient Safety & Learning System (2008). BC Patient Safety & Learning System. http://bcpslscentral.ca/wp-content/uploads/2014/04/PSLSEvaluationReport_FINAL_Jan2508_website1.pdf.

[28] Alshammari T.M., Alenzi K.A., Alatawi Y., Almordi A.S., Altebainawi A.F. (2022). Current situation of medication errors in Saudi Arabia: A nationwide observational study. J. Patient Saf..

[29] Vincent C. (2007). Incident reporting and patient safety. BMJ.

[30] Kamboj A., Spiller H.A., Casavant M.J., Chounthirath T., Hodges N.L., Smith G.A. (2018). Antidepressant and antipsychotic medication errors reported to United States poison control centers. Pharmacoepidemiol. Drug Saf..

[31] Alshehri G.H., Keers R.N., Carson-Stevens A., Ashcroft D.M. (2021). Medication safety in mental health hospitals: A mixed-methods analysis of incidents reported to the national reporting and learning system. J. Patient Saf..

[32] Tansuwannarat P., Vichiensanth P., Sivarak O., Tongpoo A., Promrungsri P., Sriapha C., Wananukul W., Trakulsrichai S. (2023). A 10-Year Retrospective Analysis of Medication Errors among Adult Patients: Characteristics and Outcomes. Pharmacy.

[33] Sirriyeh R., Lawton R., Gardner P., Armitage G. (2010). Coping with medical error: A systematic review of papers to assess the effects of involvement in medical errors on healthcare professionals’ psychological well-being. Qual. Saf. Health Care.

[34] Rodziewicz T.L., Houseman B., Vaqar S., Hipskind J.E. (2025). Medical Error Reduction and Prevention. StatPearls [Internet].

[35] Schwendimann R., Blatter C., Dhaini S., Simon M., Ausserhofer D. (2018). The occurrence, types, consequences and preventability of in-hospital adverse events—A scoping review. BMC Health Serv. Res..

[36] San Jose-Saras D., Valencia-Martín J.L., Vicente-Guijarro J., Moreno-Nunez P., Pardo-Hernández A., Aranaz-Andres J.M. (2022). Adverse events: An expensive and avoidable hospital problem. Ann. Med..

[37] Fernholm R., Holzmann M.J., Wachtler C., Szulkin R., Carlsson A.C., Pukk Härenstam K. (2020). Patient-related factors associated with an increased risk of being a reported case of preventable harm in first-line health care: A case-control study. BMC Fam. Pract..

[38] Keers R.N., Williams S.D., Cooke J., Ashcroft D.M. (2013). Causes of medication administration errors in hospitals: A systematic review of quantitative and qualitative evidence. Drug Saf..

[39] Koenig H.G., Al Zaben F., Sehlo M.G., Khalifa D.A., Al Ahwal M.S. (2013). Current state of psychiatry in Saudi Arabia. Int. J. Psychiatry Med..

[40] Qureshi N.A., Al-Habeeb A.A., Koenig H.G. (2013). Mental health system in Saudi Arabia: An overview. Neuropsychiatr. Dis. Treat..

[41] Al-Habeeb A.A., Qureshi N.A., Al-Maliki T.A. (2012). Pattern of child and adolescent psychiatric disorders among patients consulting publicly-funded child psychiatric clinics in Saudi Arabia. East. Mediterr. Health J..

[42] Koenig H.G., Al Zaben F., Sehlo M.G., Khalifa D.A., Al Ahwal M.S., Qureshi N.A., Al-Habeeb A.A. (2014). Mental health care in Saudi Arabia: Past, present and future. Open J. Psychiatry.

[43] World Health Organization (2021). Global Patient Safety Action Plan 2021–2030: Towards Eliminating Avoidable Harm in Health Care.

[44] Chowdhury S., Mok D., Leenen L. (2021). Transformation of health care and the new model of care in Saudi Arabia: Kingdom’s Vision 2030. J. Med. Life.

[45] Parentela G., Alharbi H., Alahmadi A., Alburkani H., Aljumayi I. (2019). Socio-demography and psychosis symptom severity among male schizophrenia—Diagnosed patients of MOH Mental Health Facilities, Kingdom of Saudi Arabia; A correlational study. Arch. Psychiatr. Nurs..

[46] Safiri S., Noori M., Nejadghaderi S.A., Shamekh A., Sullman M.J., Collins G.S., Kolahi A.-A. (2024). The burden of schizophrenia in the Middle East and North Africa region, 1990–2019. Sci. Rep..

[47] Semahegn A., Torpey K., Manu A., Assefa N., Tesfaye G., Ankomah A. (2020). Psychotropic medication non-adherence and its associated factors among patients with major psychiatric disorders: A systematic review and meta-analysis. Syst. Rev..

[48] Benchimol E.I., Smeeth L., Guttmann A., Harron K., Moher D., Petersen I., Sørensen H.T., von Elm E., Langan S.M., Committee R.W. (2015). The REporting of studies Conducted using Observational Routinely-collected health Data (RECORD) statement. PLoS Med..

[49] Carson-Stevens A., Hibbert P., Avery A., Butlin A., Carter B., Cooper A., Evans H.P., Gibson R., Luff D., Makeham M. (2015). A cross-sectional mixed methods study protocol to generate learning from patient safety incidents reported from general practice. BMJ Open.

[50] World Health Organization & WHO Patient Safety (2010). Conceptual Framework for the International Classification for Patient Safety, Version 1.1: Final Technical Report January 2009 (WHO/IER/PSP/2010.2).

[51] Bonheur A.N., Philips K., Hametz P., Choi J., Xie X., Soshnick S.H., Cabana M.D., Cassel-Choudhury G. (2025). Incident reporting and harmful safety events during the COVID-19 pandemic in a children’s hospital. BMC Res. Notes.

[52] Al Meslamani A.Z. (2023). Medication errors during a pandemic: What have we learnt?. Expert Opin. Drug Saf..

[53] Wysocki V., Grabe D., Meek P. (2022). SA44 Trends in Medication Error Reporting during the COVID-19 Pandemic: An Analysis of Faers Data, 2016 to 2021. Value Health.

[54] Almazrou D., Egunsola O., Ali S., Bagalb A. (2021). Medication misadventures among COVID-19 patients in Saudi Arabia. Cureus.

[55] McGrath J., Saha S., Chant D., Welham J. (2008). Schizophrenia: A concise overview of incidence, prevalence, and mortality. Epidemiol. Rev..

[56] Lawton R., Parker D. (2002). Barriers to incident reporting in a healthcare system. BMJ Qual. Saf..

[57] Sarvadikar A., Prescott G., Williams D. (2010). Attitudes to reporting medication error among differing healthcare professionals. Eur. J. Clin. Pharmacol..

[58] Meltzer H.Y., Gadaleta E. (2021). Contrasting typical and atypical antipsychotic drugs. Focus.

[59] Wright P., O’Flaherty L. (2003). Antipsychotic drugs: Atypical advantages and typical disadvantages. Ir. J. Psychol. Med..

[60] Alkhadhari S., Al Zain N., Darwish T., Khan S., Okasha T., Ramy H., Tadros T.M. (2015). Use of second-generation antipsychotics in the acute inpatient management of schizophrenia in the Middle East. Neuropsychiatr. Dis. Treat..

[61] Khawagi W.Y., Steinke D.T., Nguyen J., Pontefract S., Keers R.N. (2021). Development of prescribing safety indicators related to mental health disorders and medications: Modified e-Delphi study. Br. J. Clin. Pharmacol..

[62] Ascher-Svanum H., Zhu B., Faries D., Landbloom R., Swartz M., Swanson J. (2006). Time to discontinuation of atypical versus typical antipsychotics in the naturalistic treatment of schizophrenia. Bmc Psychiatry.

[63] Stubbs J., Haw C., Taylor D. (2006). Prescription errors in psychiatry–a multi-centre study. J. Psychopharmacol..

[64] Stubbs J., Haw C., Cahill C. (2004). Auditing prescribing errors in a psychiatric hospital. Are pharmacists’ interventions effective?. Hosp. Pharm..

[65] Tabatabaee S.S., Ghavami V., Javan-Noughabi J., Kakemam E. (2022). Occurrence and types of medication error and its associated factors in a reference teaching hospital in northeastern Iran: A retrospective study of medical records. BMC Health Serv. Res..

[66] Rothschild J.M., Mann K., Keohane C.A., Williams D.H., Foskett C., Rosen S.L., Flaherty L., Chu J.A., Bates D.W. (2007). Medication safety in a psychiatric hospital. Gen. Hosp. Psychiatry.

[67] Khawagi W.Y., Steinke D., Carr M.J., Wright A.K., Ashcroft D.M., Avery A., Keers R.N. (2022). Evaluating the safety of mental health-related prescribing in UK primary care: A cross-sectional study using the clinical practice research Datalink (CPRD). BMJ Qual. Saf..

[68] Khoja T., Neyaz Y., Qureshi N.A., Magzoub M.A., Haycox A., Walley T. (2011). Medication errors in primary care in Riyadh City, Saudi Arabia. East Mediterr Health J..

[69] Alsaleh F.M., Alsaeed S., Alsairafi Z.K., Almandil N.B., Naser A.Y., Bayoud T. (2021). Medication errors in secondary care hospitals in Kuwait: The perspectives of healthcare professionals. Front. Med..

[70] Caspi H., Perlman Y., Westreich S. (2023). Managing near-miss reporting in hospitals: The dynamics between staff members’ willingness to report and management’s handling of near-miss events. Saf. Sci..

[71] Ayre M.J., Lewis P.J., Phipps D.L., Keers R.N. (2023). unDerstandIng the cauSes of mediCation errOrs and adVerse drug evEnts for patients with mental illness in community caRe (DISCOVER): A qualitative study. Front. Psychiatry.

[72] Avery A.J., Rodgers S., Cantrill J.A., Armstrong S., Cresswell K., Eden M., Elliott R.A., Howard R., Kendrick D., Morris C.J. (2012). A pharmacist-led information technology intervention for medication errors (PINCER): A multicentre, cluster randomised, controlled trial and cost-effectiveness analysis. Lancet.

[73] Onder G., Van Der Cammen T.J., Petrovic M., Somers A., Rajkumar C. (2013). Strategies to reduce the risk of iatrogenic illness in complex older adults. Age Ageing.

[74] Krähenbühl-Melcher A., Schlienger R., Lampert M., Haschke M., Drewe J., Krähenbühl S. (2007). Drug-related problems in hospitals: A review of the recent literature. Drug Saf..

[75] Khalil H., Kynoch K., Hines S. (2020). Interventions to ensure medication safety in acute care: An umbrella review. JBI Evid. Implement..

[76] Sawamura K., Ito H., Yamazumi S., Kurita H. (2005). Interception of potential adverse drug events in long-term psychiatric care units. Psychiatry Clin. Neurosci..

[77] Ito H., Yamazumi S. (2003). Common types of medication errors on long-term psychiatric care units. Int. J. Qual. Health Care.

[78] Paoletti R.D., Suess T.M., Lesko M.G., Feroli A.A., Kennel J.A., Mahler J.M., Sauders T. (2007). Using bar-code technology and medication observation methodology for safer medication administration. Am. J. Health-Syst. Pharm..

[79] Chapuis C., Roustit M., Bal G., Schwebel C., Pansu P., David-Tchouda S., Foroni L., Calop J., Timsit J.-F., Allenet B. (2010). Automated drug dispensing system reduces medication errors in an intensive care setting. Crit. Care Med..

[80] Langan S.M., Schmidt S., Wing K., Ehrenstein V., Nicholls S., Filion K., Klungel O., Petersen I., Sorensen H., Guttmann A. (2018). The REporting of studies Conducted using Observational Routinely-collected health Data (RECORD) Statement for Pharmacoepidemiology (RECORD-PE). BMJ.

