# Supplementary material for: Exploring Medication Errors with Antipsychotics in Saudi Arabia: Insights from a Nationwide Analysis
Source: Healthcare (Basel). 2025 Oct 27;13(21):2705. doi: 10.3390/healthcare13212705 (PMC12607312; doi:10.3390/healthcare13212705)
Supplement: Supplementary file 1 [file healthcare-13-02705-s001.zip › Supplementary File 2.pdf]

Supplementary File S2

Table S2: List of antipsychotic medications according to the Ministry of Health formulary

**Antipsychotics**
Olanzapine
Quetiapine
Haloperidol
Risperidone
Aripiprazole
Amisulpride
Clozapine
Chlorpromazine
Flupenthixol
Trifluoperazine
Sulpride
Zuclopenthixol
Paliperidone
Fluphenazine
